# Perinatal Environmental Risks for Eosinophilic Esophagitis: A Systemic Review and Meta-Analysis

**DOI:** 10.7759/cureus.66326

**Published:** 2024-08-06

**Authors:** Nicha Wongjarupong, Malique Delbrune, Busara Songtanin, Erin E Reardon, Daphne M Moutsoglou, Vikram J Christian, Joshua A Sloan

**Affiliations:** 1 Division of Gastroenterology, Hepatology, and Nutrition, Department of Medicine, University of Minnesota, Minneapolis, USA; 2 Department of Medicine, University of Minnesota School of Medicine, Minneapolis, USA; 3 Department of Medicine, Texas Tech University Health Sciences Center Lubbock, Lubbock, USA; 4 Health Sciences Library, University of Minnesota, Minneapolis, USA; 5 Division of Gastroenterology, Hepatology, and Nutrition, Department of Medicine, Minneapolis VA Health Care System, Minneapolis, USA; 6 Department of Medicine, Gastroenterology Section, Minneapolis VA Health Care System, Minneapolis, USA; 7 Division of Pediatric Gastroenterology, Hepatology and Nutrition, Department of Pediatrics, University of Minnesota, Minneapolis, USA

**Keywords:** antibiotic, cesarean section, eosinophilic esophagitis, risk factor, preterm

## Abstract

There are limited data on the association of eosinophilic esophagitis (EoE) and environmental risk factors. The aim of this study was to determine the potential associations between perinatal risk factors and EoE.

A search was conducted for relevant studies published up to December 12th, 2023, using MEDLINE, EMBASE, Scopus, Web of Sciences, and Cochrane databases. Risk ratios with the 95% confidence interval (CI) were estimated using a random-effects model. Case-control or cohort studies that determined perinatal environmental factors within the first year of life and their association with EoE were included.

Six case-control studies were included in the analysis. Six studies (2,087 EoE and 6,786 controls) were included for risk of infant antibiotic use with a pooled risk ratio of 1.30 (95%CI: 1.11-1.52, I2 = 76%), and five studies were included for cesarean section with a pooled risk ratio of 1.22 (95%CI: 1.10-1.34, I2 = 5%). There were three studies for breastfeeding with a pooled risk ratio of 1.07 (95%CI: 1.00-1.15, I2 = 0%); five studies were included for preterm birth with a pooled risk ratio of 1.52 (95%CI: 1.14-2.04, I2 = 48%). There were three studies for neonatal intensive care unit admission with a pooled risk ratio of 1.75 (95% CI: 1.41-2.18, I2 = 0%). Publication bias was found between EoE and infant antibiotic use and cesarean section, but not for EoE and preterm birth, neonatal care unit admission, or breastfeeding.

This meta-analysis suggests a weak association between antibiotic use during the first year of life, cesarean section, preterm birth, and neonatal intensive care unit admission and a possible risk of EoE. Further studies are warranted to confirm these findings as they may be indirect associations rather than causal.

## Introduction and background

Introduction

Eosinophilic esophagitis (EoE) is an immune-mediated process triggered by food antigens or aeroallergens in the setting of impaired esophageal epithelial barrier resulting in T-helper 2-mediated inflammation [1]. The increased prevalence of EoE for the past 20 years suggests possible environmental factors as a part of pathophysiology [2]. Along with increased underlying prevalence, the recognition of symptoms and endoscopic appearance has increased. The prevalence of EoE reported in a large US database was approximately six in 10,000 persons [3]. Age of onset is variable and clinical presentation varies by age. Children under the age of 18 years old account for 24% of cases [3]. In adults, dysphagia is common, while children may present with failure to thrive, gastroesophageal reflux, dysphagia, or food impaction [4].

Atopic diseases including asthma, allergic rhinitis, and eczema have been shown to be associated with EoE [5]. Additionally, atopic illnesses have been related to perinatal risk factors including cesarean sections and early-age antibiotic use, suggesting a possible correlation between EoE and perinatal risk factors [5]. Previous case-control studies have demonstrated associations between perinatal factors and EoE including infant antibiotic use, cesarean section, formula feeding, and preterm birth [6-11].

Understanding the EoE pathophysiology and its associated risk factors will help improve the management and prevention of EoE to further decrease the disease healthcare burden. Patients with EoE have significantly decreased health-related quality of life and increased healthcare utilization [12,13]. In 2019 in the United States, 14% of patients with EoE required emergency department visits, of which nearly 40% required an upper endoscopy for food impaction [13]. The aim of this systematic review and meta-analysis is to determine the association between perinatal risk factors and EoE.

## Review

Methods

Data Sources and Search Strategy

We performed a systematic review as per PRISMA (Preferred Reporting Items for Systematic Reviews and Meta-analyses) guidelines. PRISMA guidelines consisted of a checklist of 27 items used to guide the full capturing and inclusion of necessary information for the systematic review [14]. We searched six databases including MEDLINE via Ovid, EMBASE via Ovid, Scopus, Web of Science Core Collection, Cochrane Library, and ClinicalTrials.gov for full-text studies and conference abstracts published from the inception of the databases through December 12, 2023. Abstracts found in these databases containing key phrases for the search were then manually reviewed by the team for determination of eligibility criteria. The search strategy is provided in Supplementary 1 data (Appendices). References of the included studies were reviewed for other potentially relevant studies. The study protocol was registered with PROSPERO (International Prospective Register of Systematic Reviews; no. CRD42022332329).

Study Selection and Eligibility Criteria

Two authors (NW and BS) independently screened titles and abstracts of all the identified studies for eligibility. Disagreements were identified and discussed with the senior author (JS). The case-control or cohort studies that determined risk factors associated with EoE were assessed for full-text availability. Full-text studies were included if the study met all of the following criteria: (i) case-control or cohort study; (ii) EoE, defined with histopathology exam, ICD-9, or ICD-10 codes, as the outcome of interest; (iii) perinatal environmental factors within the first year of life including route of delivery, preterm birth, neonatal intensive care unit (NICU) admission, breastfeeding, and antibiotic use during the first year of life; (iv) provided adequate information for calculation of pooled relative risk. Studies were excluded if there was inadequate information for the analysis including crude numbers of EoE patients and controls. If more than one study had the same patient cohort, the study with the higher quality score assessed by the Newcastle-Ottawa Scale was included. The corresponding authors of each study were contacted for additional data and information if needed.

Infant antibiotic use was defined as any record of antibiotic use during the first year of life. The route of delivery was classified as either cesarean section or vaginal delivery. Preterm birth was defined as gestational age at birth of less than 37 weeks. Breastfeeding was defined as any breastfeeding during a lifetime. NICU admission was defined as any NICU admission during the first year of life.

Data Extraction

Data were independently extracted from full-text articles by two authors (NW and MD). Disagreements were identified and discussed with the senior author (JS). The information extracted included: the diagnostic criteria of EoE, the crude number of individuals with and without perinatal risk factors in the EoE and control groups, the source of controls (population-based or hospital-based), the number of participants, the country where the study was conducted, and publication year. If there were inadequate data available, the corresponding authors were contacted via email to provide further additional information.

Data Synthesis and Analysis

The pooled risk ratio and 95% confidence interval (CI) of each perinatal risk factor were calculated from the crude number of the study patients and controls using a random-effect model. The random-effect model was used because of the significant heterogeneity of the included studies. Both the I2 statistics and p-value were used to assess the heterogeneity among studies. An I2 value of >50% or p-value <0.1 indicates substantial heterogeneity. Publication bias was assessed by Funnel plots. If there were an adequate number of studies, subgroup analyses were conducted based on study quality. Analyses were performed using Review Manager (RevMan) version 5.4 (Copenhagen, Denmark).

Quality Assessment

The quality of case-control studies was assessed by the Newcastle-Ottawa Scale (NOS), with three sections: selection (up to four points), comparability (up to two points), and outcome (up to three points), with a maximum of nine points. The study quality was classified as good (score 7-9), fair (score 4-6), or poor (score 0-3) [15].

Results

Study Characteristics

**Figure 1:**
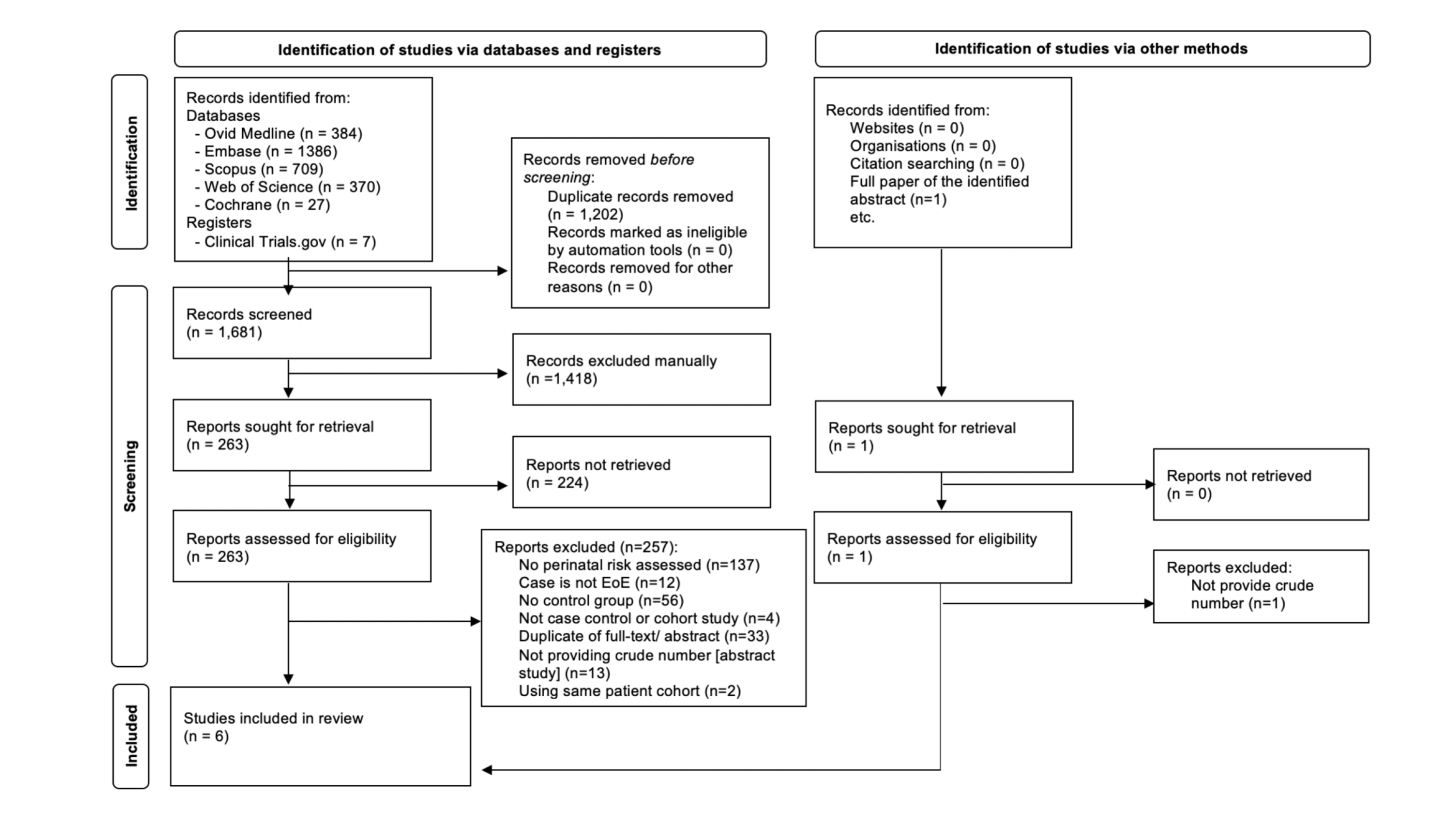
PRISMA 2020 flow diagram for new systematic reviews which included searches of databases, registers, and other sources PRISMA: Preferred Reporting Items for Systematic Reviews and Meta-analyses; EoE: eosinophilic esophagitis

From the electronic search with the removal of duplicates, 1,681 studies were identified (Figure 1). After the abstract screening, 263 studies involving risk factors for EoE were further assessed for full text.

There were 10 studies that met all inclusion criteria. However, four of the 10 studies were excluded. The excluded studies included three studies with the same patient cohort including Jensen et al., 2018 [16], which was similar to another publication from the same author of Jensen et al., 2018 [17]; Daniels et al., 2018 [18], which was similar to Witmer et al., 2018 [19]; and Kurt et al., 2023 [20], which was similar to Jensen et al., 2023 [21]. Radano et al., 2014 [9] were excluded as we were unable to obtain the required additional information to include in the analysis.

There were two studies that were conducted at the University of North Carolina. Jensen et al., 2013 [22] and Dellon et al., 2021 [23], which were identified as not having overlapping patients. Jensen et al., 2013 [22] was a study of pediatric patients who had EoE from 2004-2010, whereas Dellon et al., 2021 [23] was a prospective study that included adult patients with a new diagnosis of EoE from 2009 to 2015.

Finally, six case-control studies were included for the final analyses [10,17,19, 21-23]. There were no cohort studies included. The time period ranged from 1997 to 2018. There was a total of 2,087 cases of patients with EoE and 6,786 controls (Table 1). Four studies were conducted in the United States [17,19,22,23]. One study was conducted in Denmark [21], and one study was conducted in Canada [10]. Five studies were hospital-based case-control studies [10,17,22,23], while the other two studies were a population-based case-control study [21] and a national database case-control study, respectively [19]. The diagnostic criteria of EoE of each study were provided in Supplementary Data 2. Regarding the study quality per the Newcastle-Ottawa Scale, three studies were rated as good quality [17,19,21], and three studies were rated as fair quality [10,22,23] (Supplementary data 3).

**Table 1:** Characteristics of eight case-control studies of perinatal environmental factors and eosinophilic esophagitis (EoE) EoE: eosinophilic esophagitis; Ped: pediatric; EGD: esophagogastroduodenoscopy

Author, Year, Country, State	Study Year	Adult/Ped	Source of EOE/Control	Number of EoE/control	Matched by	Assessment or Exposure	Study Quality
Jensen et al., 2013 USA, NC [22]	2004-2010	Ped	Hospital/Community, siblings of plastic surgery patients	26/31	Age	Mother questionnaire	5, Fair
Slae et al., 2015 Canada [10]	N/A	Ped	Hospital/Hospital, patients underwent EGD	102/167	N/A	Parent questionnaire	5, Fair
Jensen et al., 2018 USA, OH [17]	N/A	Ped	Hospital/Population	1410/2820	Sex, age, time period	Parent questionnaire	7, Good
Witmer et al., 2018 USA [19]	2008-2015	Ped	Hospital/Population, military health database	127/ 121	Time period	ICD-9	9, Good
Dellon et al., 2021 USA, NC [23]	2009-2015	Adult	Hospital/Population	40/40	None	Mother questionnaire	6, Fair
Jensen et al., 2023 Denmark [21]	Birth year of 1997-2018	Ped	Population/Population, national database	392/ 3637	Age, sex	Prescription data	9, Good

Infant antibiotics use (within the first 1 year) and association with EoE

**Figure 2:**
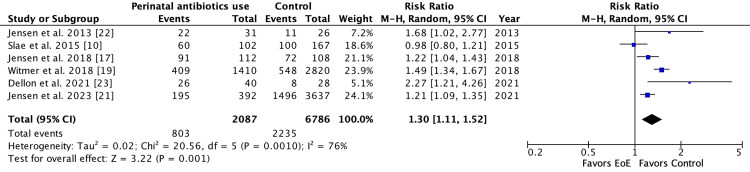
Forest plot of studies showing the risk ratio of the association between infant antibiotic use and EoE EoE: eosinophilic esophagitis

There were six studies (2,087 EoE patients and 6,786 controls) included for the risk of infant antibiotic use. All except Witmer et al., 2018 defined the infant antibiotic use of any antibiotic exposure during the first 12 months of life [10,17,21-23]. Witmer et al. (2018) defined infant antibiotic use as any antibiotic exposure during the first six months of life [19]. Of the six studies, four studies found a statistically significant positive association between infant antibiotic use [19,21-23]. The pooled risk ratio for EoE was 1.30 (95%CI: 1.11-1.52) with statistically significant heterogeneity among studies (P=0.0010, I2 = 76%, Figure 2).

Other perinatal risk factors and association with EoE

**Figure 3:**
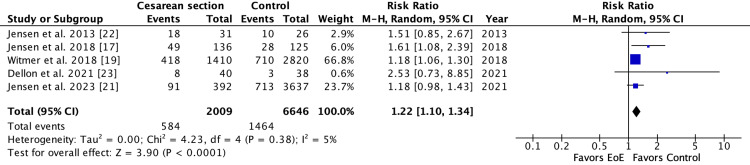
Forest plot of studies showing the risk ratio of the association between cesarean section and EoE EoE: eosinophilic esophagitis

**Figure 4:**
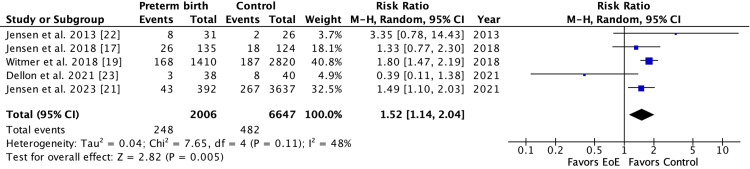
Forest plot of studies showing the risk ratio of the association between preterm birth and EoE EoE: eosinophilic esophagitis

**Figure 5:**
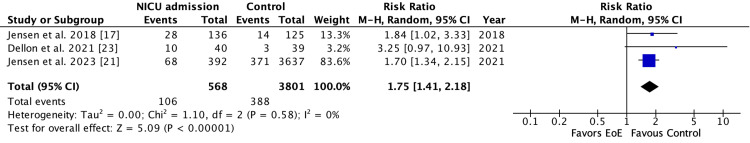
Forest plot of studies showing the risk ratio of the association between neonatal intensive care unit admission and EoE EoE: eosinophilic esophagitis

**Figure 6:**
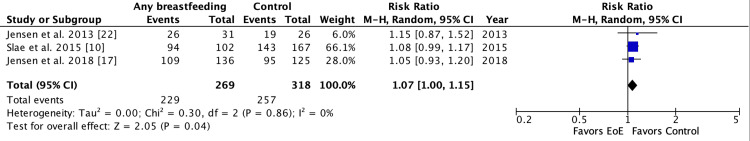
Forest plot of studies showing the risk ratio of the association between breastfeeding and EoE EoE: eosinophilic esophagitis

Five studies (2,009 EoE patients and 6,646 controls) were included for cesarean section with a pooled risk ratio of 1.22 (95%CI: 1.10-1.34, I2 = 5%, P=0.38,) (Figure 3) [17,19,21-23]. There were five studies (2,006 EoE patients and 6,647 controls) included for preterm birth with a pooled risk ratio of 1.52 (95%CI: 1.14-2.04, I2 = 48%, P=0.11) (Figure 4) [17,19,21-23]. There were three studies (568 EoE patients and 3,801 controls) found for NICU admission with a pooled risk ratio of 1.75 (95% CI: 1.41-2.18), I2 = 0%, P=0.58,) (Figure 5) [17,21,23]. There were three studies (269 EoE patients and 318 controls) for breastfeeding with a pooled risk ratio of 1.07 (95%CI: 1.00-1.15, I2 = 0%, P=0.86) (Figure 6) [10,17,22]. There were only two studies (133 EoE patients and 193 controls) for exclusive breastfeeding with a pooled risk ratio of 0.81 (95%CI: 0.24-2.70, I2 = 63%, P=0.10) [17].

Publication bias

**Figure 7:**
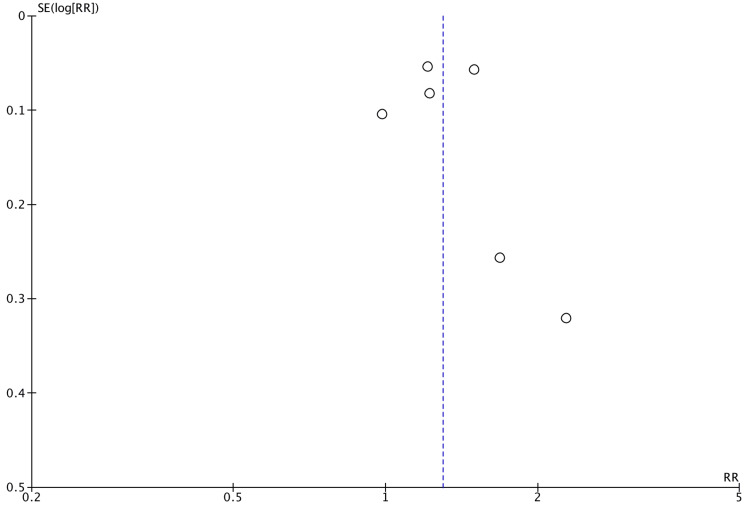
Funnel plot of publication bias for association between infant antibiotic use and EoE EoE: eosinophilic esophagitis

**Figure 8:**
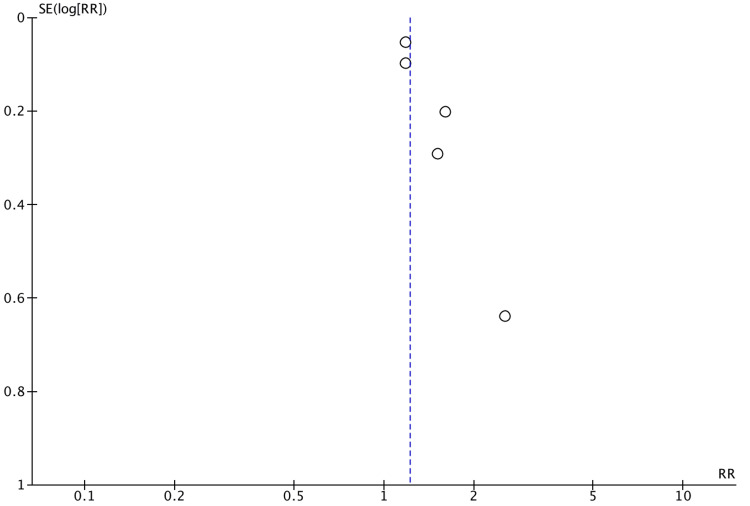
Funnel plot of publication bias for association between cesarean section and EoE EoE: eosinophilic esophagitis

**Figure 9:**
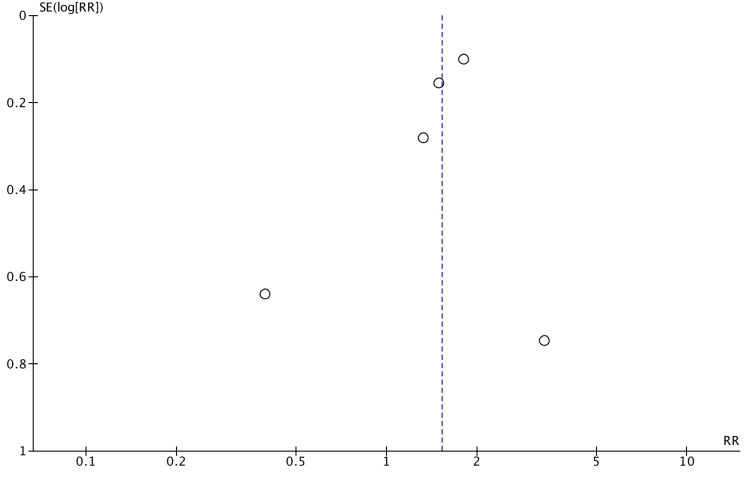
Funnel plot of publication bias for the association between preterm delivery use and EoE EoE: eosinophilic esophagitis

Publication bias was found with larger studies reporting positive associations between infant antibiotic use and EoE, as well as between cesarean section and EoE (Figures 7-8). No bias was determined for the association of preterm delivery (Figure 9). There were a limited number of included studies for NICU admission and breastfeeding. The Egger’s regression asymmetry test was not conducted due to a limited number of included studies.

Discussion

This meta-analysis of six case-control studies suggests a possible association between EoE and infant antibiotic use, cesarean section, NICU admission, and preterm birth.

Prior studies have identified possible microbiome changes in patients with EoE [24]. The gut microbiome of EoE patients has reduced diversity and altered taxonomy including reduced relative abundances of specific bacteria such as Firmicutes and Clostridiales [24]. Clostridia-containing microbiota have been shown to promote regulatory T-cell expansion and immunoglobulins [25]. Bacterial production of anti-inflammatory short-chain fatty acids by bacterial members of the phylum Firmicutes also promotes regulatory T-cell activity [26]. Regulatory T cells promote tolerance and inhibit T helper 2 cells and atopic immune responses [26]. Recapitulating these findings, pediatric esophageal microbiomes show a higher abundance of Proteobacteria and lower abundances of Firmicutes in patients with EoE compared to non-EoE pediatric controls [24,27].

Antibiotic use during the prenatal period and early life has been associated with an increased risk of other atopic conditions, including atopic dermatitis [28-30]. Microbiota significantly shape the infant’s immune system early in life with possible lasting effects into adulthood by promoting tolerance [31]. In a murine model, antibiotics promoted inflammation by inducing translocation of live, intact bacteria [31]. In humans, bacterial translocation may promote inflammation, reducing tolerance to oral antigens.

We found cesarean section and EoE to be associated. Cesarean section is associated with allergic rhinitis, asthma, and food allergy [32,33]. The proposed mechanism is through altered gut microbiomes through lack of contact with the mother’s vaginal and fecal bacteria during delivery. In this regard, vaginal delivery may act as a natural form of microbiota transplant therapy. Supporting this, a prior study found that there were taxonomic differences between three-month-old infants delivered by cesarean section versus vaginal delivery [34]. Cesarean delivery reduces fecal microbiome diversity and reduces circulating T helper 1 cytokines [31], both of which could promote a T helper 2 cell environment and decrease regulatory T cell responses [35].

Infants with a history of NICU admission tend to have more comorbidities, and also higher concurrent antibiotics use with prior cross-sectional data of up to 20% prevalence of antibiotic use of infants admitted in the NICU [36]. Similarly, preterm births are more likely to be exposed to antibiotics with 7% in preterm and 2% in term delivery [36]. However, there is no clear evidence apart from the included studies in this meta-analysis that infants with higher comorbidities had a higher incidence of EoE.

More research is needed to definitively determine if microbiome changes during antibiotic use and cesarean section promote EoE, or if changes are a consequence of the condition. These findings provide a plausible link between the microbiome and possible downstream immune effects that may mediate disease and argue for further study. There are several limitations in this meta-analysis. First, there was heterogeneity among the included studies with wide variation in size. Second, the elapsed time since exposure could contribute to recall bias in the patients in the hospital-based studies. Lastly, more studies are needed to explore risk factors such as breastfeeding and EoE development.

## Conclusions

This meta-analysis suggests there were weak associations of antibiotic use during the first year of life, cesarean section, preterm birth, and NICU admission with a risk of EoE development. With the available literature at this time, there is no association between breastfeeding and the risk of developing EoE. While it is unknown if the perinatal factors are related to microbiome changes, and if microbiome changes seen in pediatric EoE patients are causative or a consequence of the condition, this meta-analysis provides a plausible link between the microbiome and possible downstream immune effects that may mediate disease and argue for further study. Ultimately, more research is needed to further elucidate the risk factors associated with the development of EoE as well as the potential microbiome involvement.

## Appendices

Supplementary data 1: Searching strategy

All search strategies are from the inception of the databases through December 12, 2023.

Ovid Medline (384)

1: exp Eosinophilic Esophagitis/ or (exp Eosinophilia/ and exp Esophagitis/) -- 2984

2: ((eosinophil* adj2 esophagit*) or (eosinophil* adj2 oesophagit*)).ti,ab,kw,kf. -- 3940

3: 1 or 2 --4184

4: exp Environmental Exposure/ or exp Risk Factors/ or exp Premature Birth/ or exp "Infant, Premature"/ or exp "Obstetric Labor, Premature"/ or exp Breast Feeding/ or exp Cesarean Section/ or (exp Infant/ and exp Anti-Bacterial Agents/) or (exp Pregnancy/ and exp "Diet, Food, and Nutrition"/) or exp Maternal Nutritional Physiological Phenomena/ or exp Birth Weight/ or exp "Infant, Low Birth Weight"/ or exp "Intensive Care Units, Neonatal"/ or (exp Infant/ and exp Proton Pump Inhibitors/) or exp Population/ or exp Population Health/ --1770766

5: ((environment* adj2 (expos* or factor* or risk*)) or exposome* or (((gestat* or prenatal or intrapartum or postnatal or pregnan* or early life or infan*) adj2 (expos* or factor* or risk*)) or (risk adj2 factor*)) or ((preterm or prematur*) adj2 (birth* or labor* or infant* or neonat*)) or (breastfeed* or breast feed* or breast fed or breastfed or wet nurs*) or (((cesarean or caesarean or postcesarean) adj2 section*) or (abdominal adj2 deliver*) or c-section*) or ((infant* or baby or babies or neonat*) and (((anti-bacterial or antibacterial or bacteriocidal or anti-mycobacterial or antimycobacterial) adj2 (agent* or compound*)) or antibiotic* or bacteriocide*)) or ((mother or maternal* or pregnan* or prenatal) adj2 (eat* or feed* or nutrition* or nutrient* or vitamin*)) or (birth weight or low-birth-weight or (low adj2 birth weight)) or (((neonat* or newborn) adj2 (intensive care unit* or ICU*)) or NICU*) or ((infant* or baby or babies or neonat*) and ((proton adj2 pump adj2 inhibit*) or Dexlansoprazole or Esomeprazole or Lansoprazole or Omeprazole or Pantoprazole or Rabeprazole or scopadulciol or timoprazole or xanthoangelol or salvianolic acid or cassigarol)) or (urban or rural or suburban or city or cities)).ti,ab,kw,kf. --1669234

6: 4 or 5 --2708928

7: 3 and 6 --389

8: exp animals/ --26811101

9: exp humans/ --21634853

10: 8 not 9 --5176248

11: 7 not 10 --384

Embase (1386)

1: exp Eosinophilic Esophagitis/ or (exp Eosinophilia/ and exp Esophagitis/) --9763

2: ((eosinophil* adj2 esophagit*) or (eosinophil* adj2 oesophagit*)).ti,ab,kw,kf. --8396

3: 1 or 2 --10291

4: exp Environmental Exposure/ or exp Risk Factor/ or exp prematurity/ or exp premature labor/ or exp Breast Feeding/ or exp Cesarean Section/ or (exp Infant/ and exp antibiotic agent/) or (exp Pregnancy/ and exp nutrition/) or exp maternal nutrition/ or Birth Weight/ or exp low birth weight/ or exp neonatal intensive care unit/ or (exp Infant/ and exp proton pump inhibitor/) or exp Population/ or exp Population Health/ --2636245

5: ((environment* adj2 (expos* or factor* or risk*)) or exposome* or (((gestat* or prenatal or intrapartum or postnatal or pregnan* or early life or infan*) adj2 (expos* or factor* or risk*)) or (risk adj2 factor*)) or ((preterm or prematur*) adj2 (birth* or labor* or infant* or neonat*)) or (breastfeed* or breast feed* or breast fed or breastfed or wet nurs*) or (((cesarean or caesarean or postcesarean) adj2 section*) or (abdominal adj2 deliver*) or c-section*) or ((infant* or baby or babies or neonat*) and (((anti-bacterial or antibacterial or bacteriocidal or anti-mycobacterial or antimycobacterial) adj2 (agent* or compound*)) or antibiotic* or bacteriocide*)) or ((mother or maternal* or pregnan* or prenatal) adj2 (eat* or feed* or nutrition* or nutrient* or vitamin*)) or (birth weight or low-birth-weight or (low adj2 birth weight)) or (((neonat* or newborn) adj2 (intensive care unit* or ICU*)) or NICU*) or ((infant* or baby or babies or neonat*) and ((proton adj2 pump adj2 inhibit*) or Dexlansoprazole or Esomeprazole or Lansoprazole or Omeprazole or Pantoprazole or Rabeprazole or scopadulciol or timoprazole or xanthoangelol or salvianolic acid or cassigarol)) or (urban or rural or suburban or city or cities)).ti,ab,kw,kf. --2334063

6: 4 or 5 --3723571

7: 3 and 6 --1403

8: exp animal/ not exp human/ --5970753

9: 7 not 8 --1386

Scopus (709)

( ( ( INDEXTERMS ( "Eosinophilic Esophagitis"  OR  ( "Eosinophilia"  AND  "Esophagitis" ) ) )  OR  ( TITLE-ABS-KEY (((eosinophil* W/2 esophagit*) or (eosinophil* W/2 oesophagit*))) ) )  AND  ( ( INDEXTERMS ( "Environmental Exposure"  OR  "Risk Factors"  OR  "Premature Birth"  OR  "Infant, Premature"  OR  "Obstetric Labor, Premature"  OR  "prematurity"  OR  "premature labor"  OR  "Breast Feeding"  OR  "Cesarean Section"  OR  ( "Infant"  AND  ( "Anti-Bacterial Agents"  OR  "antibiotic agent" ) )  OR  ( "Pregnancy"  AND  ( "Diet, Food, and Nutrition"  OR  "nutrition" ) )  OR  "Maternal Nutritional Physiological Phenomena"  OR  "maternal nutrition"  OR  "Birth Weight"  OR  "Infant, Low Birth Weight"  OR  "low birth weight"  OR  "Intensive Care Units, Neonatal"  OR  "neonatal intensive care unit"  OR  ( "Infant"  AND  ( "Proton Pump Inhibitors"  OR  "proton pump inhibitor" ) )  OR  "Population"  OR  "Population Health" ) )  OR  ( TITLE-ABS-KEY ( ( ( environment*  W/2  ( expos*  OR  factor*  OR  risk* ) )  OR  exposome* )  OR  ( ( ( gestat*  OR  prenatal  OR  intrapartum  OR  postnatal  OR  pregnan*  OR  "early life"  OR  infan* )  W/2  ( expos*  OR  factor*  OR  risk* ) )  OR  ( risk  W/2  factor* ) )  OR  ( ( preterm  OR  prematur* )  W/2  ( birth*  OR  labor*  OR  infant*  OR  neonat* ) )  OR  ( breastfeed*  OR  "breast feed*"  OR  "breast fed"  OR  breastfed  OR  "wet nurs*" )  OR  ( ( ( cesarean  OR  caesarean  OR  postcesarean )  W/2  section* )  OR  ( abdominal  W/2  deliver* )  OR  c-section* )  OR  ( ( infant*  OR  baby  OR  babies  OR  neonat* )  AND  ( ( ( anti-bacterial  OR  antibacterial  OR  bacteriocidal  OR  anti-mycobacterial  OR  antimycobacterial )  W/2  ( agent*  OR  compound* ) )  OR  antibiotic*  OR  bacteriocide* ) )  OR  ( ( mother  OR  maternal*  OR  pregnan*  OR  prenatal )  W/2  ( eat*  OR  feed*  OR  nutrition*  OR  nutrient*  OR  vitamin* ) )  OR  ( "birth weight"  OR  low-birth-weight  OR  ( low  W/2  birth  AND weight ) )  OR  ( ( ( neonat*  OR  newborn )  W/2  ( "intensive care unit*"  OR  icu* ) )  OR  nicu* )  OR  ( ( infant*  OR  baby  OR  babies  OR  neonat* )  AND  ( ( proton  W/2  pump  W/2  inhibit* )  OR  dexlansoprazole  OR  esomeprazole  OR  lansoprazole  OR  omeprazole  OR  pantoprazole  OR  rabeprazole  OR  scopadulciol  OR  timoprazole  OR  xanthoangelol  OR  salvianolic  AND acid  OR  cassigarol ) )  OR  ( urban  OR  rural  OR  suburban  OR  city  OR  cities ) ) ) ) )  AND NOT  ( ( ( ( INDEXTERMS ( adult  OR  adolescent  OR  child  OR  infant ) )  AND NOT  ( INDEXTERMS ( adult ) ) )  OR  ( ( INDEXTERMS ( animals  OR  animal ) )  AND NOT  ( INDEXTERMS ( humans  OR  human ) ) ) ) )

Web of Science (370)

Indexes=SCI-EXPANDED, SSCI, A&HCI, CPCI-S, CPCI-SSH, BKCI-S, BKCI-SSH, ESCI, CCR-EXPANDED, IC Timespan=All years

(((TS=((eosinophil* NEAR/2 esophagit*) or (eosinophil* NEAR/2 oesophagit*)))) AND ((TS=(((environment* NEAR/2 (expos* or factor* or risk*)) or exposome*) or (((gestat* or prenatal or intrapartum or postnatal or pregnan* or "early life" or infan*) NEAR/2 (expos* or factor* or risk*)) or (risk NEAR/2 factor*)) or ((preterm or prematur*) NEAR/2 (birth* or labor* or infant* or neonat*)) or (breastfeed* or "breast feed*" or "breast fed" or breastfed or "wet nurs*") or (((cesarean or caesarean or postcesarean) NEAR/2 section*) or (abdominal NEAR/2 deliver*) or c-section*) or ((infant* or baby or babies or neonat*) and (((anti-bacterial or antibacterial or bacteriocidal or anti-mycobacterial or antimycobacterial) NEAR/2 (agent* or compound*)) or antibiotic* or bacteriocide*)) or ((mother or maternal* or pregnan* or prenatal) NEAR/2 (eat* or feed* or nutrition* or nutrient* or vitamin*)) or ("birth weight" or low-birth-weight or (low NEAR/2 birth weight)) or (((neonat* or newborn) NEAR/2 ("intensive care unit*" or ICU*)) or NICU*) or ((infant* or baby or babies or neonat*) and ((proton NEAR/2 pump NEAR/2 inhibit*) or Dexlansoprazole or Esomeprazole or Lansoprazole or Omeprazole or Pantoprazole or Rabeprazole or scopadulciol or timoprazole or xanthoangelol or salvianolic acid or cassigarol)) or (urban or rural or suburban or city or cities)))))

Cochrane (27)

#1: MeSH descriptor: [Eosinophilic Esophagitis] explode all trees --182

#2: MeSH descriptor: [Eosinophilia] explode all trees --646

#3: MeSH descriptor: [Esophagitis] explode all trees --1149

#4: #2 AND #3 --189

#5: (eosinophil* NEAR/2 esophagit*) OR (eosinophil* NEAR/2 oesophagit*) --480

#6: #1 or #4 or #5 --480

#7: MeSH descriptor: [Environmental Exposure] explode all trees --3563

#8: MeSH descriptor: [Risk Factors] explode all trees --33304

#9: MeSH descriptor: [Premature Birth] explode all trees --2174

#10: MeSH descriptor: [Infant, Premature] explode all trees --4977

#11: MeSH descriptor: [Obstetric Labor, Premature] explode all trees --2964

#12: MeSH descriptor: [Breast Feeding] explode all trees --2697

#13: MeSH descriptor: [Cesarean Section] explode all trees --4697

#14: MeSH descriptor: [Infant] explode all trees --42247

#15: MeSH descriptor: [Anti-Bacterial Agents] explode all trees --15337

#16: #14 and #15 --1542

#17: MeSH descriptor: [Pregnancy] explode all trees --31666

#18: MeSH descriptor: [Diet, Food, and Nutrition] explode all trees --74008

#19: #17 and #18 --3719

#20: MeSH descriptor: [Maternal Nutritional Physiological Phenomena] explode all trees --541

#21: MeSH descriptor: [Birth Weight] explode all trees --2571

#22: MeSH descriptor: [Infant, Low Birth Weight] explode all trees --2661

#23: MeSH descriptor: [Infant] explode all trees --42247

#24: MeSH descriptor: [Proton Pump Inhibitors] explode all trees --1806

#25: #23 and #24 --43

#26: MeSH descriptor: [Population] explode all trees --10030

#27: MeSH descriptor: [Population Health] explode all trees --1003

#28: #7 or #8 or #9 or #10 or #11 or #12 or #13 or #16 or #19 or #20 or #21 or #22 or #25 or #26 or #27 --66874

#29: ((environment* NEAR/2 (expos* or factor* or risk*)) or exposome* or (((gestat* or prenatal or intrapartum or postnatal or pregnan* or "early life" or infan*) NEAR/2 (expos* or factor* or risk*)) or (risk NEAR/2 factor*)) or ((preterm or prematur*) NEAR/2 (birth* or labor* or infant* or neonat*)) or (breastfeed* or "breast feed*" or "breast fed" or breastfed or "wet nurs*") or (((cesarean or caesarean or postcesarean) NEAR/2 section*) or (abdominal NEAR/2 deliver*) or c-section*) or ((infant* or baby or babies or neonat*) and (((anti-bacterial or antibacterial or bacteriocidal or anti-mycobacterial or antimycobacterial) NEAR/2 (agent* or compound*)) or antibiotic* or bacteriocide*)) or ((mother or maternal* or pregnan* or prenatal) NEAR/2 (eat* or feed* or nutrition* or nutrient* or vitamin*)) or ("birth weight" or low-birth-weight or (low NEAR/2 "birth weight")) or (((neonat* or newborn) NEAR/2 ("intensive care unit*" or ICU*)) or NICU*) or ((infant* or baby or babies or neonat*) and ((proton NEAR/2 pump NEAR/2 inhibit*) or Dexlansoprazole or Esomeprazole or Lansoprazole or Omeprazole or Pantoprazole or Rabeprazole or scopadulciol or timoprazole or xanthoangelol or "salvianolic acid" or cassigarol)) or (urban or rural or suburban or city or cities)) --186496

#30: #28 OR #29 --195577

#31: #6 AND #30 --27

ClinicalTrials.gov (7)

Condition or Disease: Eosinophilic Esophagitis

Other terms: (risk factor*) OR (environmental factor*) OR (environmental risk) OR (environmental exposure*) OR (early life factor*) OR (early life risk) OR (early life exposure*) OR (prenatal factor*) OR (prenatal risk*) OR (prenatal exposure*) OR (infant risk*)

Supplementary data 2: Diagnosis criteria for EoE of the included studies

1. Jensen et al., 2013 [22]: Esophageal eosinophil >15 HPF, not responsive to PPI

2. Slae et al., 2015 [10]: Esophageal eosinophil >15 HPF, not responsive to PPI

3. Jensen et al., 2018 [17]: ICD-9

4. Witmer et al., 2018 [19]: Esophageal eosinophil >15 HPF

5. Dellon et al, 2021 [23]: Esophageal eosinophil >15 HPF, not responsive to PPI

6. Jensen et al., 2023 [21]: Esophageal eosinophilia on biopsy, no other associated condition to esophageal eosinophilia

Supplementary data 3: Study quality assessment with Newcastle-Ottawa Scale

Scores including Selection (4), Comparability (2), Exposure (3), and Total score (9)

1. Jensen et al., 2013 [22]: 4, 0, 1, 5, total 5 (Fair)

2. Slae et al., 2015 [10]: 3, 0, 2, total 5 (Fair)

3. Jenson et al., 2018 [17]: 4, 1, 3, total 7 (Good)

4. Witmer et al., 2018 [19]: 4, 2, 3, total 9 (Good)

5. Dellon et al., 2021 [23]: 4, 0, 2, total 6 (Fair)

6. Jensen et al., 2023 [21]: 4, 2, 3, total 9 (Good)
